# Overview of REJOIN: A Clinical Trial to Use Exercise to Relieve Joint Pain in Older Breast Cancer Survivors

**DOI:** 10.1093/geroni/igaa057.2239

**Published:** 2020-12-16

**Authors:** Shirley Bluethmann, Cristina Truica, Nancy Olsen, Christopher Sciamanna, Heidi Klepin, Vernon Chinchilli, Kathryn Schmitz

**Affiliations:** 1 Penn State College of Medicine, Hershey, Pennsylvania, United States; 2 Penn State Cancer Institute, Hershey, Pennsylvania, United States; 3 Wake Forest School of Medicine, Winston-Salem, North Carolina, United States

## Abstract

Aromatase Inhibitors (AIs) are recommended for survival in hormone-sensitive breast cancer survivors, yet are underutilized, especially in older survivors. Joint pain is a prevalent AI-related symptom that is associated with low adherence. The aims for this randomized clinical trial (n=76) are to: 1) adapt a self-management (exercise + education) intervention for older survivors planning to take AIs; 2) Test the effect of a pilot intervention on arthralgia; and 3) Test its effect on AI medication adherence behaviors. We will adapt a program for seniors that includes bi-weekly 60-minute sessions of supervised exercise plus 30 minutes of education. The 16-week program includes: 8-weeks in person plus 8-weeks at home with phone counseling. We will conduct geriatric plus baseline assessment of exercise, joint pain, and AI adherence (repeated 4, 6 and 12 months). More research with geriatric survivors is required to address treatment needs and to promote survival. Part of a symposium sponsored by the Cancer and Aging Interest Group.

